# Huge solitary gallstone complicated with liver cirrhosis in a middle-aged woman: case report

**DOI:** 10.1097/MS9.0000000000000296

**Published:** 2023-07-28

**Authors:** Agapiti Hipoliti Chuwa

**Affiliations:** Department of Medical Physiology, Mbeya College of Health and Allied Sciences, University of Dar es Salaam, Mbeya, Tanzania

**Keywords:** Cholelithiasis, cirrhosis, Klatskin tumour, FOX01, case report

## Abstract

**Background:**

Gallstones are increasingly becoming a common diagnosis in hospitals across the continents, with predominance in women. Patients suspected of a gallstone disease require thorough evaluation including endoscopic ultrasound, magnetic resonance imaging, or magnetic resonance cholangiography. A delayed or missed diagnosis is associated with serious complications and poor prognosis.

**Case presentation:**

A 44-year-old female patient presented with fever, vomiting, hypochondria, and epigastric pain for 10 days. Clinical examination showed jaundice and tenderness at the right hypochondriac region. Blood analysis revealed elevated bilirubin, alkaline phosphatase, and white blood cells. The patient was sent for a computed tomography (CT) scan which showed a grossly enlarged liver about 17.2 cm in length and a hypo-attenuating mass in the gallbladder fossa that enhanced moderately and heterogeneously following intravenous contrast administration. Dilated intrahepatic biliary ducts were also appreciated. Explorative laparotomy was performed and revealed an enlarged, cirrhotic-appearing liver, a thickened gallbladder, and a whitish-yellow gallstone about 3 cm in the largest diameter situated at its neck. No isolated tumour was found.

**Clinical discussion:**

Although gallstone disease is very common, misdiagnosis still occurs especially in low and lower-middle-income countries. Inadequate evaluation and increased utilization of CT in emergency and surgical departments are the contributing factors for a missed diagnosis.

**Conclusions:**

A missed gallstone disease occurs due to various factors including inappropriate standard operating procedures, which set a CT scan as the first imaging test for all internal conditions. This case report presents the appropriate approach to achieving the diagnosis of a gallstone disease before surgical intervention.

## Introduction

Highlights

- Misdiagnosis of gallstone disease is common. This case presents a missed gallstone disease and discusses how to appropriately carry out a diagnostic workout to achieve the diagnosis.
- Biliary tumours can have signs and symptoms mimicking those of a gallstone disease. This report describes how to radiologically distinguish cholangiocarcinomas and gallstones to allow for correct planning of management.
- Evidence indicates an increased utilization of computed tomography in emergency and surgical departments. This report highlights the effects of over utilization of computed tomography in surgical departments as a contributing factor to missed diagnoses and unnecessary surgical operations which may worsen the patient’s prognosis.

Gallstone disease is increasingly becoming a common diagnosis. Two major types of gallstones have been described namely: cholesterol and pigment stones. Cholesterol gallstones account for more than 75% of all gallstone diagnoses in western countries^1^ and are usually caused by local factors such as stasis, crystallization of cholesterol, and excessive secretion of mucin^2^, and systemic factors such as the interleukins-6, interleukins-7, interleukins-12, and interleukins-13, and other pro-inflammatory cytokines^3,4^. Molecular pathways involving the activity of HMG-hydroxyl-methyl-glutaryl coenzyme A (CoA) reductase, the transcription factor forkhead box protein 01 (FOX01), and the expression of ATP-binding cassette transporters G5 and G8 (ABCG5 and ABCG8) genes have also been described in the pathogenesis of gallstones^5,6^.

Symptomatic gallstone disease may present with some typical features, however, confirmation of the diagnosis requires thorough evaluation. Abdominal ultrasonography is the imaging test of choice with a specificity of more than 95%^7^. When ultrasound findings are inconclusive, an MRI and endoscopic ultrasound are recommended^7,8^. The use of a computed tomography (CT) is less useful for the diagnosis of gallstones, hence, should be discouraged.

This case report has been reported in line with the SCARE 2020 criteria^9^.

### Patient information

A 44-year-old obese, non-pregnant, African female patient presented to the surgical department with a 10-day history of fever, and sharp pain in the epigastric and right hypochondriac regions. The patient reported episodes of nausea, vomiting, and altered bowel movements. The patient was on antibiotics and analgesics for 10 days for cholecystitis without improvement. Her past surgical history was insignificant.

### Clinical findings

On clinical examination, the patient appeared sick, however, afebrile, jaundiced, and had a body mass index of 32. The blood pressure was 130/85 mmHg, pulse rate was 80 beats per min and body temperature was 37.1^o^C. Abdominal examination revealed an enlarged tender liver.

### Diagnostic assessment

**Figure 1:**
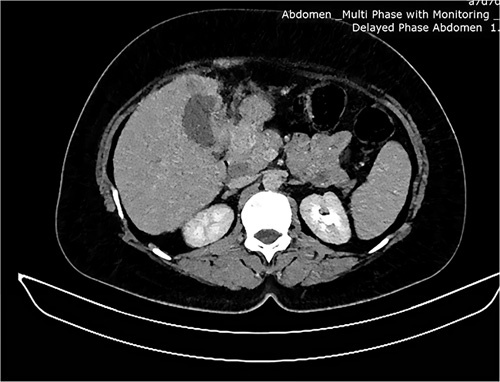
A computed tomography image showing enlarged liver and gallbladder mass.

Laboratory investigation showed an elevated total white blood cell count of 12 000 cells/mm^3^, elevated alkaline phosphatase 786 IU/l, and a bilirubin level of 2 mg/dl. The patient was put under 64-slice multidetector CT where serial axial sections were recorded, and a grossly enlarged liver measuring 17.2 cm in length was described (Figure 1). Poorly marginalized hypo-attenuating mass was demonstrated in the gallbladder fossa with extension to the adjacent segment IV measuring 7.2×6.5×5.3 cm. The mass was enhanced moderately and heterogeneously following intravenous contrast administration. Dilated intrahepatic biliary ducts were also appreciated. The common bile duct was not dilated and there was no evidence of calculus. The rest of the abdominal organs were essentially normal. A conclusion was that features were suggestive of a gallbladder tumour with hepatic extension and obstructive intrahepatic biliary dilatation, which was differentiated from a Klatskin tumour.

### Therapeutic intervention

**Figure 2:**
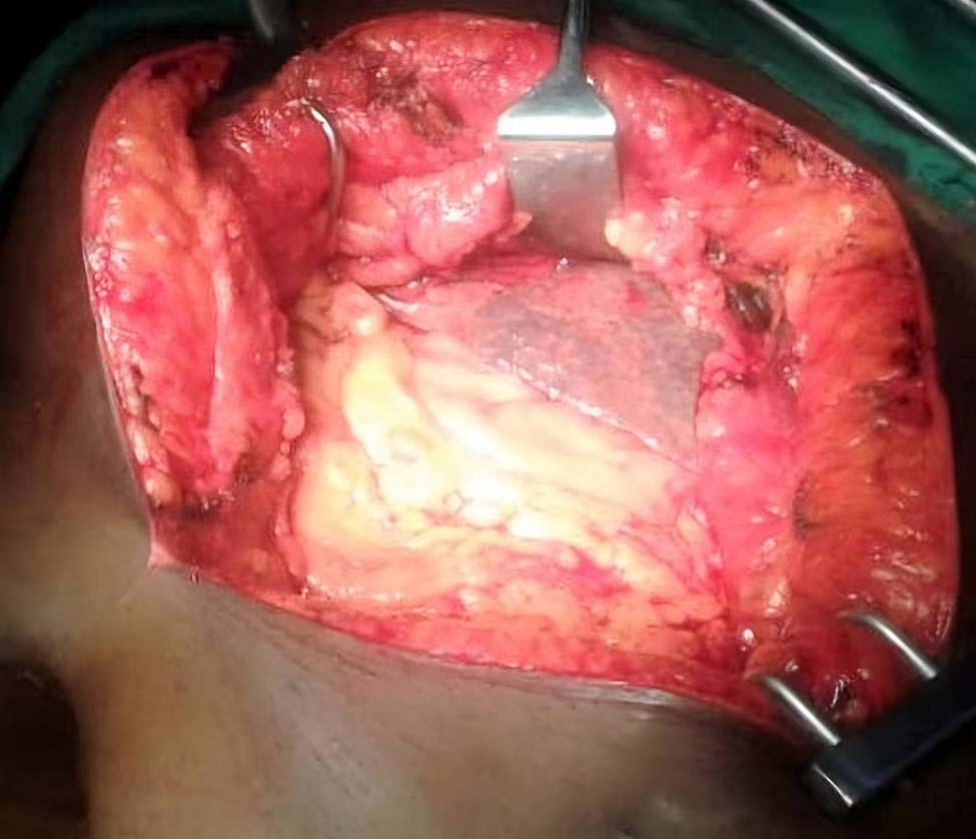
Nodules seen on the liver surface during exploratory laparotomy.

**Figure 3:**
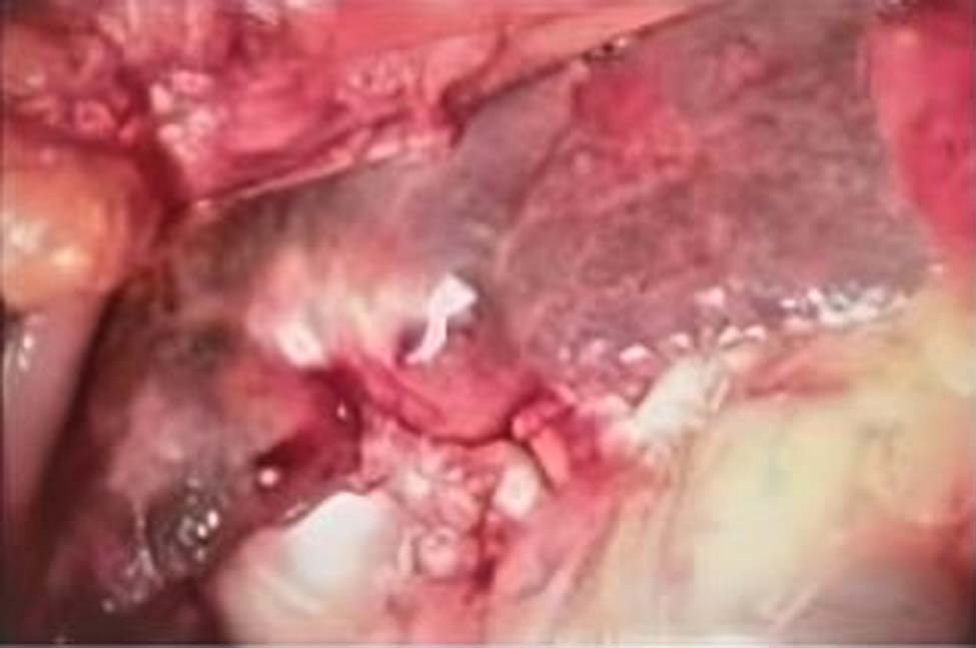
Cirrhotic changes seen on the liver surface indicating chronic liver injury.

**Figure 4:**
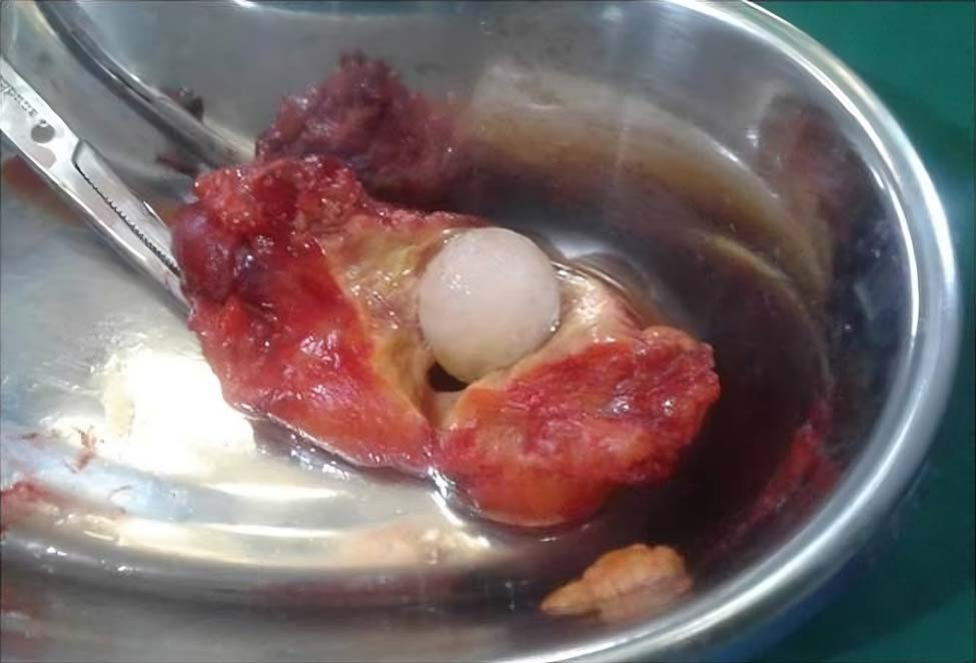
Excised gallbladder revealing a massively thickened wall and a gallstone.

**Figure 5:**
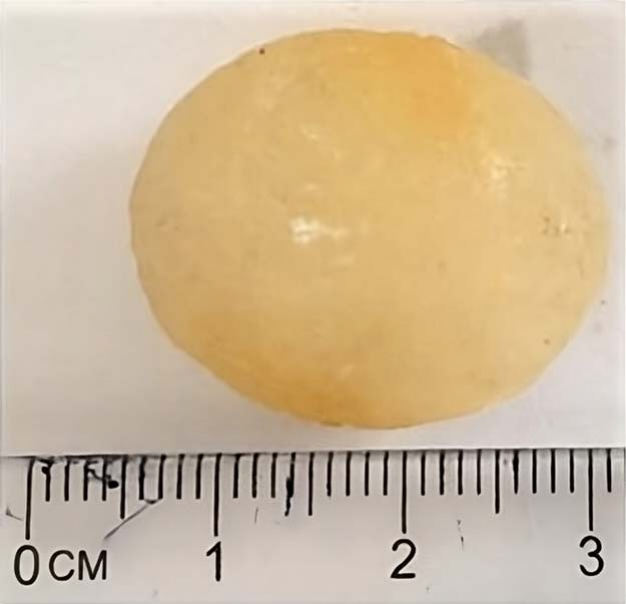
Huge whitish-yellow gallstone extracted from the gallbladder.

A laparotomy was performed by a general surgeon with 5 years of experience and the liver was exposed, which showed multiple small nodules on its surface; suggesting cirrhotic changes, Figures 2 and 3. Explorative cholecystectomy was performed with the extraction of a huge whitish-yellow solitary stone from its lumen. The gallbladder wall was thickened and mimicked a tumour, (Figures 4 and 5) however, no isolated tumour was found. Sections of tissue taken from the liver and gallbladder were sent for histopathology and the results showed vascularized fibrotic septa linking portal tracts with central veins creating what is called hepatocyte islands. These islands lacked central veins.

### Follow-up

The patient was admitted to the hospital for 12 days and was kept on systemic antibiotics, and she is set to attend a hepatology clinic every 4–6 months for periodic evaluation and for supportive treatment. A long-term prognosis could not be ascertained.

## Discussion

This report presented the findings of a huge gallstone and thickened gallbladder wall which was missed after being confused with a Klatskin tumour under a CT scan. Small gallbladder stones are, in most cases (about 80%), asymptomatic. For the symptomatic proportion (about 20%), patients usually have bigger stones and present with typical radiating pain, biliary colic, and a history of using analgesia^10^. The presence of these symptoms with definitive risk factors (female sex and increased BMI) should warrant thorough and appropriate evaluation. Since most gallbladder stones are radiolucent, abdominal ultrasonography is considered the gold standard test for the diagnosis of gallstone disease^7^. Ultrasounds are inexpensive, easy to use, and available at most health facilities, even in low and lower-middle-income countries, and have sensitivity and specificity exceeding 95% and 96%, respectively. The European Association for the Study of Liver (EASL) Clinical Practice Guidelines on the prevention, diagnosis, and treatment of gallstones^11^ require the use of endoscopic ultrasound and, or MRI when ultrasound findings are inconclusive.

Accumulating evidence suggests an increased utilization of CT in emergency departments (ED) and surgical departments in recent years; especially for privately insured patients. Bellolio *et al.* ^12^ investigated the trends in CT utilization and found a 60% increase in CT use by the ED since 2010 in all age groups, except the children. The number of annual ED visits remained stable. In a similar study conducted elsewhere^13^, increased CT utilization was strongly associated with higher costs, longer hospital stays, and emergency operations. Since CT is often considered superior to ultrasound, patients who receive a CT scan first are less likely to undergo ultrasonography for further evaluation; and when a CT is less useful, as in the case of gallstone disease, a missed diagnosis is likely.

Klatskin tumour (cholangiocarcinoma) is a rare, small, poorly differentiated tumour that arises from the epithelium of bile ducts, mostly at the bifurcation of the common hepatic duct. Symptomatic biliary tumours are, in most cases, associated with poor prognosis. Extensive workout with an multidetector CT, MRI, and, or MR cholangiography is therefore critical to define the size, location, vascular invasion, and optimal treatment strategy before surgery can be carried out^14^. Unlike in gallstone disease, direct invasion of a tumour to the liver or adjacent vessels, intrahepatic segmental biliary dilatation, and an abrupt transition between dilated and non-dilated bile ducts, periductal thickening and endoluminal lesions are diagnostic features of a Klatskin tumour in radiography. A helical computed tomography can correctly detect and localize the tumour mass in over 90% of the cases^15^.

## Conclusions

Increased utilization of CT in recent years in surgical departments is associated with increased emergency operations and the likelihood of missed diagnoses in cases where a CT is less useful; such as in the case of gallstone disease. Based on the findings in this report, we, therefore, recommend for optimization of standard operating procedures in diagnostic radiology in line with the current guidelines and protocols and emphasize a thorough diagnostic workout for patients suspected of a gallstone disease before surgery.

### Patient perspective

The patient was aware of her symptoms and was concerned about irreversible liver damage, even after the histopathology results.

## Ethical approval

None.

## Consent

Written informed consent was obtained from the patient for the publication of this case report and accompanying images. A copy of the written consent is available for review by the Editor-in-Chief of this journal on request.

## Source of funding

None.

## Author contribution

A.H.C. conceived the idea, designed the study, wrote the original manuscript, and revised the manuscript.

## Conflicts of interest disclosure

None.

## Guarantor

Agapiti Hipoliti Chuwa.

## Provenance and peer review

Not commissioned, externally peer-reviewed.
